# Spatial Quantitation of Drugs in tissues using Liquid Extraction Surface Analysis Mass Spectrometry Imaging

**DOI:** 10.1038/srep37648

**Published:** 2016-11-24

**Authors:** John G. Swales, Nicole Strittmatter, James W. Tucker, Malcolm R. Clench, Peter J. H. Webborn, Richard J. A. Goodwin

**Affiliations:** 1 Drug Safety & Metabolism, AstraZeneca R&D, Cambridge Science Park, Cambridge, CB4 0WG, UK; 2 Biomolecular Sciences Research Centre, Sheffield Hallam University, Howard Street, Sheffield S1 1WB, UK

## Abstract

Liquid extraction surface analysis mass spectrometry imaging (LESA-MSI) has been shown to be an effective tissue profiling and imaging technique, producing robust and reliable qualitative distribution images of an analyte or analytes in tissue sections. Here, we expand the use of LESA-MSI beyond qualitative analysis to a quantitative analytical technique by employing a mimetic tissue model previously shown to be applicable for MALDI-MSI quantitation. Liver homogenate was used to generate a viable and molecularly relevant control matrix for spiked drug standards which can be frozen, sectioned and subsequently analyzed for the generation of calibration curves to quantify unknown tissue section samples. The effects of extraction solvent composition, tissue thickness and solvent/tissue contact time were explored prior to any quantitative studies in order to optimize the LESA-MSI method across several different chemical entities. The use of a internal standard to normalize regional differences in ionization response across tissue sections was also investigated. Data are presented comparing quantitative results generated by LESA-MSI to LC-MS/MS. Subsequent analysis of adjacent tissue sections using DESI-MSI is also reported.

A drugs distribution and the relationship with efficacy and safety are important considerations during drug development. It is crucial that a compound is present in sufficient quantity at the site of action to deliver efficacy. Equally, excessive abundance of the drug in tissues may lead to unwanted toxicological findings rendering the drug unsafe.

Drug distribution has historically been assessed in various ways. Tissue homogenization techniques, coupled to liquid chromatography and mass spectrometry have been the mainstay to quantify drugs in tissues^1^. However, this approach results in the loss of all spatial information from the sample and merely provides an average concentration within the tissue. There is also a risk of variation caused by residual blood contamination.

Mapping and quantifying the distribution of compounds *in vivo* was historically performed using radiolabeled compounds in techniques such as quantitative whole body autoradiography (QWBA)^2^,^3^. The technique is reliable, sensitive, quantitative and retains meaningful spatial information. However, the necessity for a radiolabeled compound is a major drawback that can lead to significant ‘synthesis’ delays. Furthermore, quantitation of the parent drug can be misrepresented due to drug metabolites that still include the radiolabel.

Mass spectrometry imaging (MSI) is a complementary, viable, multiplex and label free way of elucidating the distribution of drugs and endogenous metabolites directly from the surface of tissue sections^4^. MSI is a term used to describe a group of complementary surface sampling technologies based on different mass spectrometry ionization methods. The most commonly used are matrix assisted laser desorption ionization (MALDI-MSI)^5^,^6^,^7^, secondary ion mass spectrometry (SIMS-MSI)^8^, desorption electrospray ionization (DESI-MSI)^9^,^10^ and nanoelectrospray ionization based liquid microjunction techniques such as liquid extraction surface analysis (LESA-MSI)^11^,^12^. Each technique has innate advantages and disadvantages in sensitivity, speed and spatial resolution.

MALDI-MSI and more recently DESI-MSI have been gaining popularity within the pharmaceutical industry as scientifically and economically feasible technology platforms to assess drug distribution. MALDI-MSI is capable of delivering images at low micron spatial resolution^13^, a drawback however is that it is not easily applicable to all analytes often requiring lengthy matrix optimization or on-tissue chemical derivatization to ensure detection^14^. DESI-MSI is an electrospray ionization based technique and thus has a much wider chemical scope. Spatial resolution is more limited than MALDI-MSI, typically around 50–100 μm. SIMS-MSI is again limited in chemical scope, high energy ionization and the technique being combined with low spectral resolution mass analyzers make it unsuitable for the analysis of medium and high size molecules. SIMS-MSI is capable of nanometer spatial resolution, however the technique has not been widely adopted within the pharmaceutical industry, primarily due to high set up costs and the availability of the technique within academia as a fee for service answer to bespoke distribution questions. LESA-MSI is a surface sampling technique that has been shown to be widely applicable and sensitive but can deliver only low spatial resolution images (typically 1000 μm)^11^. The technique can be combined with Orbitrap or time of flight mass spectrometers for effective use as a profiling tool^15^ but has also been used effectively with high sensitivity triple quadrupole mass spectrometers operated in selected reaction monitoring mode for mass spectrometry imaging^16^.

All of the above techniques provide relatively fast and reliable qualitative localization information that can be used to show drug distribution to target tissues or drug accumulation in tissues where a toxic effect has been observed. However this data is of a qualitative nature only and as such is often hard to put into context and difficult to draw meaningful conclusions from without a quantitative determination of the drug levels within the tissues. Quantitative MSI methods for drugs have been widely reported, most prevalently for MALDI-MSI^17^. Groseclose and Castellino published a report based on the use of tissue mimetics for the quantitation of drug compounds in liver tissue sections using MALDI-MSI^18^. Likewise, Nilsson *et al*. published MALDI-MSI data showing the quantitation of tiotropium in rat lung tissue utilizing calibration standard spotting on control tissue^19^. Other reported techniques employ the use of internal standards to normalize differences in ionization efficiency across tissue sections^20^,^21^. Quantitative methods have also been published for nano-DESI-MSI^22^ based on similar techniques as those employed in MALDI-MSI.

The research presented here details a quantitative LESA-MSI method using tissue mimetics for the quantitation of various drugs in tissue sections. Details of method optimization are reported. The quantitative LESA-MSI method was directly compared to results obtained by traditional LC-MS/MS tissue homogenization analysis and to results generated by DESI-MSI analysis.

## Methods

### Materials and reagents

Analytical grade acetonitrile, methanol and formic acid were obtained from Fisher Scientific (Loughborough, Leicestershire, UK). 2-methylbutane was obtained from Sigma-Aldrich (Poole, Dorset, UK). Test compounds were obtained in house from AstraZeneca compound management group (Macclesfield, Cheshire, UK) with the exception of moxifloxacin and SCH-23390 which were purchased from Sigma-Aldrich (Poole, Dorset, UK) and clozapine-d4 which was purchased from Qmx Laboratories (Thaxted, Essex, UK).

### Animals

Adult male Hans Wistar rats (approximate weight 260 g) were obtained from Charles River Laboratories (Margate, Kent, UK) and were acclimatized on site for a minimum of 3 days prior to dosing. Compounds were administered by oral gavage and were formulated in 5% dimethylsulfoxide/95% (30% w/v Captisol in water). Control animals were dosed with vehicle via the same administration route.

The study was performed under project license 40/3484, procedure number 10 and was reviewed and approved by the Institutional Animal Welfare and Ethical Review Body within AstraZeneca and was conducted in accordance with the animal care and ethics described in “Guidance on the Operations of the Animals (Scientific Procedures) Act 1986” issued by the UK Home Office.

### Dosing and scheduling

Liver samples were taken from 1 animal dosed with vehicle, 2 animals dosed discretely with olanzapine (10 mg/kg), and 2 cassette dosed animals (Moxifloxacin, olanzapine, erlotinib and terfenadine at 25, 10, 10 and 25 mg/kg respectively). Animals were euthanized at either 2 or 6 hrs post dose.

All tissue dissection was performed by trained AstraZeneca staff. Liver samples were harvested at termination and snap-frozen in 2-methylbutane on dry-ice, subsequent transfer of tissues was done on dry-ice and samples were stored at −80 °C until tissue processing.

### Tissue processing

Tissue sections were cryosectioned at a thickness of 14 μm and thaw mounted onto indium tin oxide (ITO) coated MALDI target slides (Bruker Daltonics, Bremen, Germany) for LESA analysis (ITO slides are not essential for LESA analysis but are used as standard within our laboratories to allow sections to be analyzed by either LESA or MALDI) and Superfrost slides (Fisher Scientific) for DESI analysis. Sections were taken at approximately equal depth from all samples. Mimetic calibration standards were thaw mounted onto the slide prior to the dosed unknowns in a semi random order to mitigate the risk of bias caused by duration on slide (mounting order - blank, 1, 20, 0.1, 5, 50, 0.5, 10, 100 nmol/g). Tissue sections from dosed and control animals were randomly thaw-mounted on the same slides (order cassette 2 h, discrete 2 h, control, cassette 6 h, discrete 6 h). Mounted tissue sections, were stored at −80 °C until analysis.

### Preparation of liver mimetics

Liver mimetics were prepared by homogenizing control liver tissue using a Fisher Powergen 500 homogenizer for a minimum of 30 s at room temperature. Each 250 μL of liver homogenate was pipetted into 9 separate molds (prepared from the bottom end of a 2 mL plastic Pasteur pipette bulb) and subsequently weighed. Appropriate volumes of compound solution (containing olanzapine, moxifloxacin, erlotinib and terfenadine) were spiked into the homogenate (assuming 1 mL of standard equivalent to 1 g of tissue and not accounting for density differences) to form liver homogenate calibration standards at 0, 0.1, 0.5, 1, 5, 10, 20, 50 and 100 nmol/g. The homogenates were then frozen at −80 °C for a minimum of 1 h prior to cryosectioning.

### LESA Optimization experiments

#### Extraction solvent composition

Three solvents were used (acetonitrile (ACN), methanol (MeOH) and isopropyl alcohol (IPA)) at 4 different compositions in water (solvent content 50, 60, 70 and 80%), each extraction solvent contained 0.1% formic acid to promote positive ionization and aide conductivity in the nanospray interface. Mimetic liver tissue containing four analytes (clozapine, albendazole, tamoxifen and astemizole) spiked at a concentration of 50 nmol/g and covering a LogD (pH 7.4) range between 2.9 and 4.09 was sectioned at a thickness of 14 μm and subsequently thaw mounted onto microscope slides. Ten LESA extractions were performed at random points across the tissue with mean signal intensity used to compare the different solvents and solvent compositions.

#### Tissue thickness

The effect of tissue thickness was assessed using mimetic liver tissue spiked with clozapine, albendazole, tamoxifen and astemizole at a concentration of 50 nmol/g and sectioned at 12, 25 and 50 μm thickness. Ten extractions were performed at random points across the tissue with mean signal intensity used to compare the differences between the sections.

#### Solvent dwell time

The effects of solvent dwell time on signal intensity were explored using mimetic liver tissue sections cut at 12, 25 and 50 μm thickness and spiked with 50 nmol/g of SCH-23390. Ten extractions were performed at random points across the tissue with mean signal intensity used to compare the differences between the sections.

#### Effects of internal standard

The effect of spraying a non-deuterated internal standard and a deuterated internal standard (IS) over the tissue to normalize differences in ionization response across different tissue areas was explored. A mimetic liver tissue calibration curve was constructed containing clozapine, albendazole, tamoxifen and astemizole (in cassette) at the following concentration levels 0, 0.1, 0.5, 1, 5, 10, 20, 50 and 100 nmol/g. A compound taken from the AstraZeneca compound collection that is routinely used as an internal standard in LC-MS/MS analysis was used as an non-deuterated IS (an acidic quinoline tetrazole, LogD 2.67, RMM 407.5), clozapine-d4 was used as a deuterated IS. The internal standard was sprayed over the tissue sections using a TMsprayer (HTx Technologies, Chapel Hill, North Carolina, USA). An additional experiment was performed with the clozapine-d4 spiked at 1 uM in the LESA extraction solvent. Ten LESA extractions were performed at random points across each calibration standard and the signal intensity was used to calculated a mean value and coefficient of variation for each calibration point in the presence and absence of the internal standard.

##### Optimized LESA quantitative mass spectrometry imaging

LESA MSI was performed using LESA-MS/MS on a Triversa Nanomate chip based electrospray ionization system (Advion, Ithaca, NY, USA) coupled to a QTRAP 5500 (AB Sciex, Framingham, MA, USA) mass spectrometer operated in positive ion MRM mode. The LESA sampling method consisted of aspiration of a 0.9 μL volume of extraction solution (Acetonitrile/water/Formic Acid 60/40/0.1 v/v/v). 0.5 μL of the solution was then dispensed at a height of 0.4 mm above the tissue with a 3 s post dispense delay time, a liquid micro junction between the pipette tip and the sample was maintained throughout the procedure. 1.1 μL of sample was re-aspirated into the pipette tip prior to infusion via the nanomate chip for MS/MS analysis. Relative abundance was determined between samples by comparison of MRM transition intensity at *m/z* 326.9 > 269.8, 266.1 > 234.3, 372.0 > 71.9 and 459.1 > 218.0 for clozapine, albendazole, tamoxifen and astemizole and 472.3 > 436.2, 313.1 > 256.1, 402.2 > 358.1 and 394.2 > 278.0 for terfenadine, olanzapine, moxifloxacin and erlotinib respectively. LESA-MS/MS data was processed using a purpose built software package capable of extracting relative abundance values from Analyst 6.1 (ABSciex, Framingham, MA, USA).

LESA-MS/MS images were created using in-house developed software capable of colour grading ion intensities acquired from each individual LESA spot in a heat map configuration fixing the colour scale at maximum for the highest intensity response in the spot and scaling the colour intensity down accordingly relative to the maximum.

### Analysis of liver homogenates by LC-MS/MS

Experimental procedures for the preparation of liver homogenates, calibration standards and quality controls and general LC-MS/MS methodologies can be found in the supplementary information.

### DESI mass spectrometry imaging

DESI MSI analysis was performed using a Q-Exactive mass spectrometer (Thermo Fisher Scientific Inc., Bremen, Germany) operated in positive ion mode. The mass spectrometer was equipped with an automated Prosolia 2D DESI source (Indianapolis, IN, USA). Mass spectra were collected in the mass range of m/z 200–600 at a mass resolution of 140,000 (at m/z 200). Methanol/water (95:5 v/v) was used as the electrospray solvent held at 4.5 kV spray voltage and delivered at a flow-rate of 1.5 μL/min by a Dionex Ultimate 3000 nanoLC pump (Thermo Fisher Scientific). Nitrogen was used as nebulising gas at a pressure of 7 bars. The height distance between the DESI sprayer and the sample surface was set to 1.5 mm with the distance between the sprayer and the inlet capillary set to 7 mm. The distance between the sample surface and the inlet capillary of the mass spectrometer was <1 mm. The angle between the sprayer tip and the sample surface was set at 750°. Spatial resolution for the imaging experiment was set to 125 μm with 173.61 μm/s scan speed at an injection time of 150 ms per spectrum. Individual horizontal line scans were combined into imzML format using the imzML converter V.1.1.4.5 (www.maldi-msi.org). Data visualization and region of interest extraction was performed using MSiReader v0.05^23^. Intensity data was extracted for each pixel within a region of interest and subsequently averaged using Microsoft Excel. All images were created using 0.01 Da bin size and linear interpolation (order 1).

## Results and Discussion

Prior to any quantitative LESA analysis a study was undertaken to optimize the method used in previously reported work from our laboratory^11^. The optimization experiments consisted of comparison of different extraction solvent systems, the effects of different tissue section thickness, the length of time the extraction solvent is in contact with the tissue (solvent dwell time) and the effects of using an internal standard on the analytical performance.

### LESA Optimization

The solvent systems tested gave varying results. Isopropyl alcohol (SI Fig. 1a) and methanol (SI Fig. 1b) based systems gave fairly low signal intensities across all of the different solvent compositions with isopropyl alcohol mixed at 60% IPA and methanol mixed at 70% MeOH giving the best results for all of the compounds tested. 70% MeOH gave signal intensities roughly 1.5-fold higher than those observed at 60% IPA. Extractions that used 70 and 80% IPA resulted in unreliable extractions 6 out of 10 times (60%), this was due to loss of the liquid junction between the tissue and the pipette tip caused by the low surface tension in high organic content solvent systems. A greater number (8/10) of extractions failed at 80% MeOH, although at all other composition ratios the number of failed extractions was less than 2/10.

Acetonitrile based solvents systems (SI Fig. 1c) gave results with higher signal intensities. Extraction failure rates at 70 and 80% ACN were 5/10 and 6/10 respectively. Failure rates at 50 and 60% ACN were zero with 60% ACN giving the highest mean signal intensity across all of the compounds and solvent compositions tested combined with a consistently good extraction. Thus, 60% ACN with 0.1% formic acid was used as a standard extraction system in all subsequent experiments.

Liver mimetic tissue sections cut at 12, 25 and 50 μm thickness were analyzed. The effect on signal intensity was negligible across the different tissue thicknesess and for each of the four compounds analyzed (SI Fig. 2). Extraction failure across each of the different sections was 3/10. It was decided to use 12 μm tissue thickness for any subsequent analysis as this is the standard used for MALDI and DESI analysis within our laboratories.

Different solvent dwell times (time solvent/tissue liquid junction is maintained) were evaluated. This showed a gradual increase for the analyte SCH-23390 when moving from 1 s to 3 s dwell time (SI Fig. 3) but the signal intensity subsequently dropped moving from the 3 s dwell time to 4 s and 5 s dwell time. Extraction failure was ≤1/10 across all of the dwell times in the experiment. Increasing variability with increasing dwell time resulted from the instability of the liquid junction over time for some extractions. A dwell time of 3 s was used as standard for all subsequent experiments.

### Effects of using an Internal Standard

The effects of using an internal standard to normalize different ionization suppression effects across tissue has been widely reported in MALDI imaging^24^,^25^. Here the most commonly used approaches were reproduced using LESA-MSI and experiments were conducted in the presence of a non-deuterated IS (standard AZ internal standard used for plasma analysis) and deuterated IS (clozapine-d4), both applied over the tissue at a constant concentration. A further experiment explored the use of the deuterated internal standard spiked into the LESA extraction solution.

When a non-deuterated IS was used the co-efficient of variation across all of the compounds at each calibration point ranged between 19.5–42.5% compared to 8.6–41.8% when no IS was used indicting greater variability when the IS was present. The differences were even more apparent when each calibration level and each compound was compared directly (SI Table 1) with the IS showing a greater co-efficient of variation at all points (with the exception of Albendazole 5 nmol/g level, 26.8% no IS vs. 24.4% with IS).

The experiment was repeated using clozapine-d4 as the IS. This had a similar result showing a clozapine co-efficient of variation range of 19.3–44.0% in the absence of IS and 17.1–64.1% in the presence of the clozapine-d4 IS. When each individual calibration point is compared (SI Table 2) the variation in the presence of the clozapine-d4 is more variable at all of the calibration points with the exception of the 5 nmol/g standard, indicating the deuterated standard made the variation worse.

As an alternative to spraying the IS over the tissue sections, spiking the IS into the LESA extraction solvent was attempted. This experiment resulted in a co-efficient of variation range of 12.8–46.3 with no IS compared to 13.0–41.1% in the presence of clozapine-d4 (SI Table 3). When individual points were compared the data with the clozapine-d4 spiked into the extraction solvent was less variable at all of the points except the 0.1 and 0.5 nmol/g calibration standards which gave 12.6% no IS vs. 19.7% with IS and 40.3% no IS vs. 41.1% with IS. This result would indicate that the presence of the deuterated internal standard decreases the variation observed in the experiment and we would recommend using this approach where a deuterated internal standard is available. Deuterated standards are not usually available in the early discovery phase of pharmaceutical development and as we intended to develop a widely applicable approach it was decided to progress the quantitation experiments without an internal standard.

### Quantitative LESA experiments

The objective of this study was to establish a robust and reproducible method for quantitative LESA-MSI analysis. The mimetic tissue model lends itself well to LESA-MSI analysis, offering a large enough sample area to accommodate the lower spatial resolution of the LESA technique, something that cannot easily be achieved using conventional standard spotting techniques used in higher spatial resolution MALDI analysis. The mimetic tissue model utilizes un-diluted homogenized tissue in the preparation of the mimetics, presenting a control matrix for spiked standards. Here we report data to support the use of mimetic tissues for quantitative LESA-MSI and compare and contrast the results to data generated by classical tissue homogenization and analysis by LC-MS/MS and to quantitative DESI-MSI.

### Quantitation of dosed rat tissues

**Figure 1:**
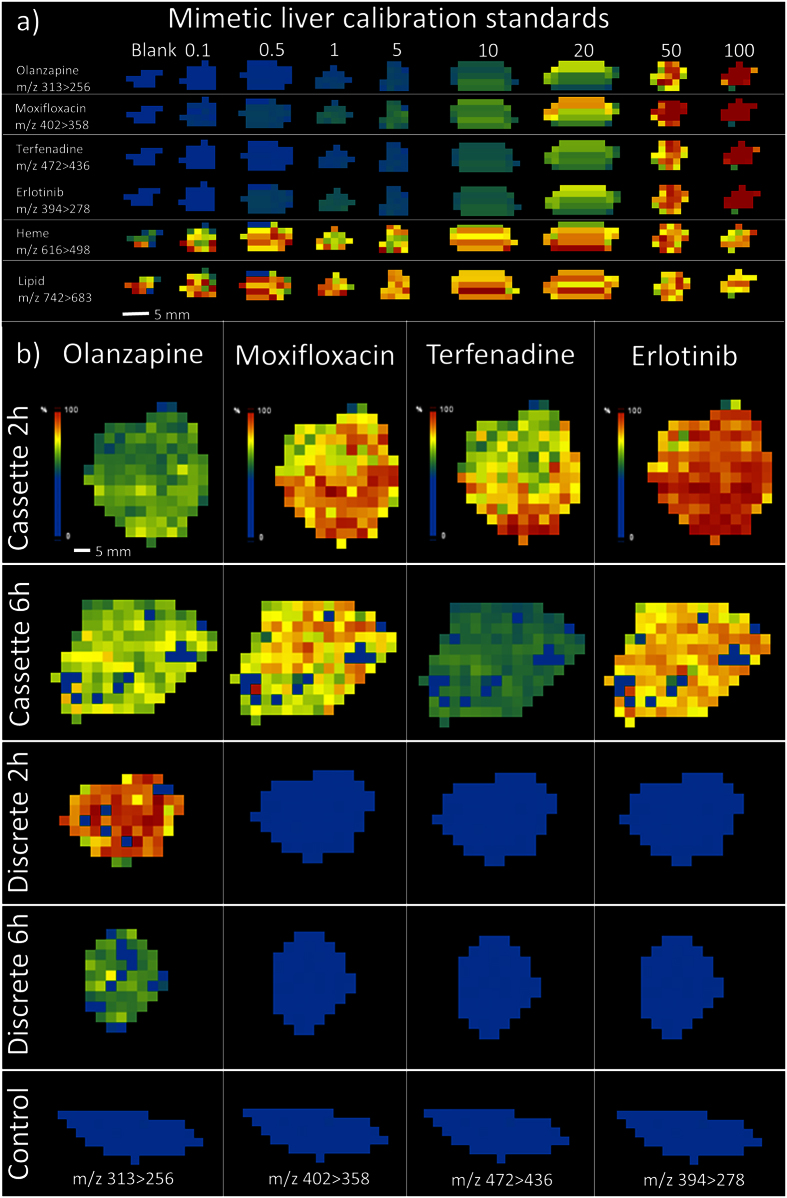
(**a**) LESA-MSI images of olanzapine, moxifloxacin, terfenadine and erlotinib in mimetic liver calibration standards showing the increasing intensity throughout the calibration range. Heme and a generic lipid marker are included to show the contrast between a spiked and endogenous response (**b**) LESA-MSI images of dosed rat liver tissue sections showing the distribution of olanazapine, moxifloxacin, terfenadine and erlotinib at 2 and 6 h post dose.

Quantitation of olanzapine, moxifloxacin, erlotinib and terfenadine was performed on liver sections were animals had been cassette dosed at 10, 25, 10, 25 mg/kg of the compounds respectively. Liver samples were collected from rats at 2 and 6 h post dose. 2 and 6 h liver sections from rats dosed discretely with olanzapine at 10 mg/kg were also tested. Residual liver tissue was sent for homogenization analysis by LC-MS/MS. Images of the mimetic liver calibration standard sections and dosed liver sections are presented in Fig. 1. The analytes were distributed continuously but at a range of abundances throughout the liver mimetics and the dosed samples. Tissue homogeneity was visually assessed via microscope after sectioning.

**Figure 2:**
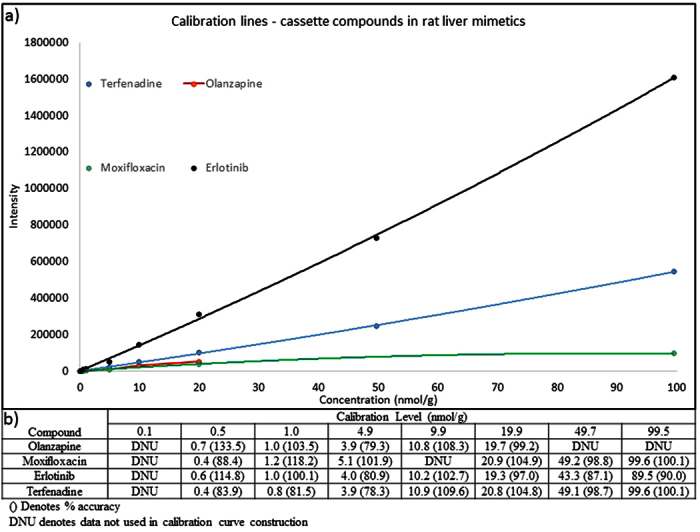
(**a**) Calibration curves of Olanzapine, moxifloxacin, terfenadine and erlotinib in rat liver mimetic tissue standards analyzed by LESA-MSI. (**b**) Back calculated concentrations of each calibration standard used in the construction of the calibration lines with corresponding accuracy values in parenthesis.

The calibration curves derived from the mimetic liver calibration standards are summarized in Fig. 2a. The linearity of the response for all four analytes was acceptable with R^2^ values equal to 0.9933, 0.9998, 0.9994 and 0.9996 for olanzapine, moxifloxacin, erlotinib and terfenadine respectively. Back calculated concentrations for all of the calibration standards used to construct the calibration curves are reported in Fig. 2b. Accuracy of the calibration standards for each compound ranged between 79.3–133.5%, 88.4–118.2%, 80.9–114.8% and 78.3–109.6% for olanzapine, moxifloxacin, erlotinib and terfenadine respectively. The LESA extraction failure rate (measured by monitoring nanospray ion current) for the experiment was ≤5%. Failed extractions were repeated at the end of the analytical run to complete the data set.

### Comparison with homogenate LC-MS/MS data

Mean intensity of each analyte in each section was calculated from all of the sampling points across the entire tissue area. This mean intensity was used to back calculate the concentration in the unknown samples from the mimetic calibration curves, giving a mean concentration across the entire tissue of interest. This value provided a direct comparison to the concentration data generated by LC-MS/MS analysis (Table 1). The concentrations observed in the unknown samples for each analyte were within the calibration range used and the correlation between the concentration data derived from LESA-MSI and LC-MS/MS was similar for each of the analytes. The percentage difference between the LESA and the LC-MS/MS results for olanzapine, moxifloxacin, erlotinib and terfenadine ranged from 9.1–32.8, 3.8–7.0, 5.6–35.1 and 14.0–32.3% respectively across the different time points and dosing regimens.

### Comparison with DESI-MSI data

**Figure 3:**
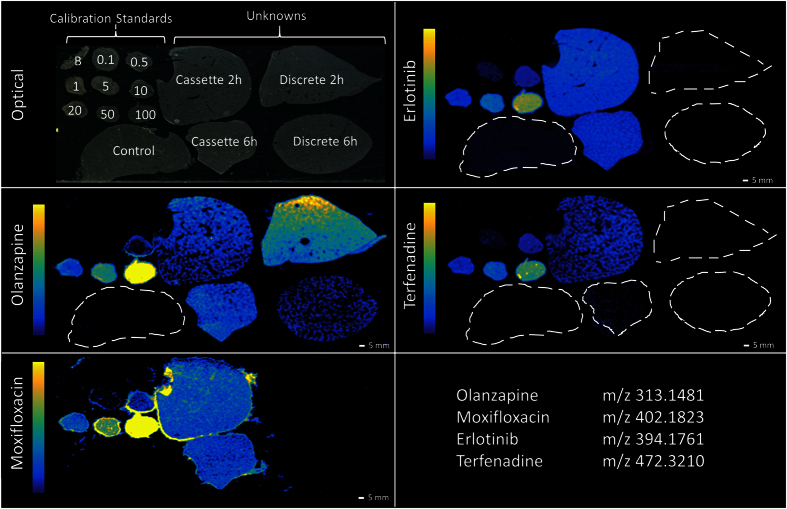
DESI-MSI images of olanzapine, moxifloxacin, erlotinib and terfenadine in rat liver mimetic calibration standards and rat liver ‘unknown’ samples. Poor detection of moxifloxacin, manifested in the compound being detected in all samples and highlighting the halo effect around the top liver mimetic tissue section.

LESA-MSI and DESI-MSI are both spray based analytical techniques and as such should show similar results. DESI-MSI is being increasingly used within academia and industry as a reliable and robust imaging technique^26^. Quantitative DESI-MSI has still to be extensively compared to other techniques such as LC-MS/MS and MALDI-MSI but several groups have reported positive quantitative measurements^22^. Adjacent sections to those run by LESA-MSI were used to repeat the analysis using DESI-MSI analysis (Fig. 3). DESI-MSI was sensitive across the calibration range used, giving a limit of quantitation (LOQ) of 1.0 nmol/g for olanzapine, erlotinib and terfenadine and 4.98 nmol/g for moxifloxacin. This compared to a LOQ of 0.5 nmol/g for all analytes by LESA-MSI analysis. To our knowledge this is the first time a comparison between the two techniques has been performed, the differences however could be caused by the use of different mass analyzers. DESI-MSI also operates at higher spatial resolution than LESA-MSI so could be inherently more sensitive when compared directly.

**Figure 4:**
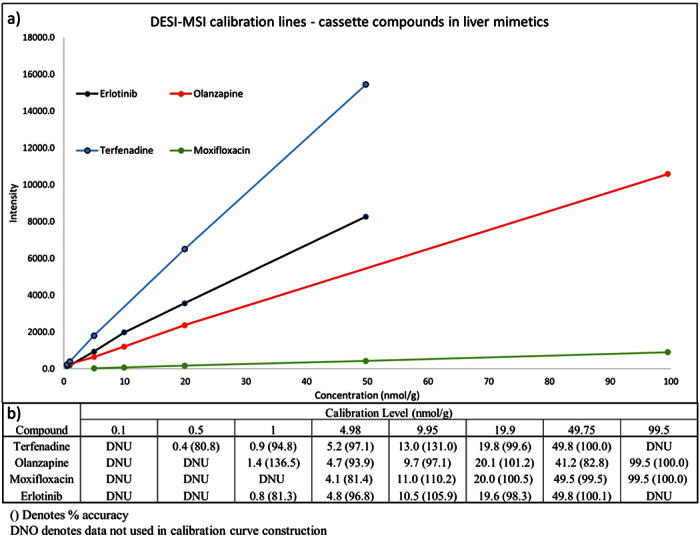
Calibration curves of Olanzapine, moxifloxacin, terfenadine and erlotinib in rat liver mimetic tissue standards analyzed by DESI-MSI. (**b**) Back calculated concentrations of each calibration standard used in the construction of the calibration lines with corresponding accuracy values in parenthesis.

The calibration curves derived from the DESI-MSI analysis of the mimetic liver calibration standards are summarized in Fig. 4a. The linearity of the response for all four analytes was good with R^2^ values equal to 0.9999, 0.9998, 0.9997 and 1.0000 for olanzapine, moxifloxacin, erlotinib and terfenadine respectively. Back calculated concentrations for all of the calibration standards used to construct the calibration curves are reported in Fig. 4b. Accuracy of the calibration standards for each compound was 82.8–136.5%, 81.4–110.2%, 81.3–105.9% and 80.8–131.0% for olanzapine, moxifloxacin, erlotinib and terfenadine respectively. Moxifloxacin sensitivity was relatively low compared to the other analytes, this can be clearly observed in Fig. 4a by the low intensity calibration curve for the compound. Sensitivity for each compound was variable between LESA-MSI and DESI-MSI with erlotinib giving the highest sensitivity in LESA-MSI but terfenadine being more sensitive in DESI-MSI. This could be due to extraction efficiency of the different compounds in the different extraction solvent systems being used in the two techniques.

The concentrations observed in the dosed samples for each analyte were within the calibration range used (Table 1). The correlation between the concentration data derived from LESA-MSI and DESI-MSI was comparable for each of the analytes. The percentage difference (using DESI-MSI as the standard) between the LESA and the DESI-MSI results for olanzapine, moxifloxacin, erlotinib and terfenadine ranged from 33.8–36.2, 26.8–44.4, 34.3–42.7 and 0.8–42.8% respectively across the different time points and dosing regimens.

DESI-MSI also correlated well with the LC-MS/MS results giving a percentage difference (using the LC-MS/MS data as standard) of 5.4–41.2%, 26.0–31.2%, 5.6–21.4% and 33.3–40.4% for olanzapine, moxifloxacin, erlotinib and terfenadine respectively.

These results were thought to be a reasonable difference when quantifying drugs in tissues using different analytical techniques.

## Conclusion

Liquid extraction surface analysis has been demonstrated to be a reliable low resolution mass spectrometry imaging technique to qualitatively image compound distribution in tissue sections. Qualitative images highlight the distribution of drugs in tissue, but often images can be misleading due to the scaling methods and any thresholding used in the visualization of the images^27^. Quantitative measurements give a definitive answer as to the drug concentration within a particular tissue. LESA-MSI has many advantages over traditional homogenization analysis such as speed of analysis, increased sample stability, low sample use allowing further analysis by other techniques and arguably the largest benefit, spatial resolution. If compound levels are located in a specific sub-compartment of an organ this information is lost with homogenization and the concentration is diluted as an average of all of the tissue sampled. Here we have shown that a mimetic tissue model lends itself well to quantitative LESA-MSI, having many advantages over more traditional quantitative mass spectrometry imaging methods. Indeed, mimetics were used as a basis for LESA method optimization in the work presented here, providing some fundamental changes that improved the sensitivity, reproducibility and robustness of the technique and ultimately a better platform on which to perform quantitative LESA analysis. The mimetic tissue can be used to make reasonably large tissue sections which makes it ideal for lower resolution imaging platforms such as LESA-MSI.

The tissue mimetic model however is not without its drawbacks. It is labor intensive when compared to simpler standard spotting techniques, uses quite high quantities of control tissue and its application to more structurally diverse tissue types such as brain has not yet been demonstrated.

LESA-MSI is often employed when other MSI techniques have failed to detect analytes of interest, due to the techniques greater sensitivity resulting from the larger sampling area. It can also be used when profiling, rather than imaging, drug distribution in large tissues from higher animals (e.g. dog liver). Typically in pharmaceutical R&D the different MSI platforms (LESA, MALDI, DESI-MSI) are used in combination, complementing data from standard bioanalysis with information from other multimodal analysis.

Quantitative LESA-MSI is not limited to detection of drugs in tissues, work is ongoing to expand the use of the mimetic model to quantitation of endogenous small molecules and small peptides as markers for drug induced efficacy in our projects using stable labelled standards.

## Additional Information

**How to cite this article**: Swales, J. G. *et al*. Spatial Quantitation of Drugs in tissues using Liquid Extraction Surface Analysis Mass Spectrometry Imaging. *Sci. Rep*. **6**, 37648; doi: 10.1038/srep37648 (2016).

**Publisher’s note:** Springer Nature remains neutral with regard to jurisdictional claims in published maps and institutional affiliations.

## Supplementary Material

Supplementary Information

## Figures and Tables

**Table 1:** Comparison of back calculated concentrations (nmol/g) for compounds analyzed by LESA-MSI, homoginization and DESI-MSI.

	Timepoint (h)											
	Cassette 2 h			Cassette 6 h			Discrete 2 h			Discrete 6 h		
Compound	LESA	Homog.	DESI	LESA	Homog.	DESI	LESA	Homog.	DESI	LESA	Homog.	DESI
Olanzapine	9.3	10.2	14.4	12.5	18.6	19.6	25.8	19.6	38.1	8.3	10.0	6.2
Moxifloxacin	15.6	14.6	10.8	12.0	12.5	16.4	—	—	—	—	—	—
Erlotinib	38.4	28.5	26.9	26.6	25.2	19.8	—	—	—	—	—	—
Terfenadine	11.9	9.0	12.0	4.0	4.7	2.8	—	—	—	—	—	—
