# Supplementary material for: Spatial Quantitation of Drugs in tissues using Liquid Extraction Surface Analysis Mass Spectrometry Imaging
Source: Sci Rep. 2016 Nov 24;6:37648. doi: 10.1038/srep37648 (PMC5121636; doi:10.1038/srep37648)
Supplement: Supplementary Information [file srep37648-s1.pdf]

Supplementary Information

Spatial Quantification of Drugs in tissues using Liquid Extraction Surface Analysis Mass Spectrometry Imaging.

John G. Swales[1, 2], Nicole Strittmatter[1], James W. Tucker[1], Malcolm R. Clench[2], Peter J.H. Webborn[1], Richard J.A. Goodwin[1]

[1] Drug Safety & Metabolism, AstraZeneca R&D, Cambridge Science Park, Cambridge, CB4 0WG, UK
[2] Biomedical Research Centre, Sheffield Hallam University, Howard Street, Sheffield S1 1WB, UK

Short title: Quantification of drugs by LESA

Keywords: LESA, DESI, MSI, Imaging

Corresponding author:

Email: john.swales@astrazeneca.com
Address: AstraZeneca, Darwin Building, Cambridge Science Park, Milton Road, Cambridge, Cambridgeshire, CB4 0WG, UK
Tel. +44(0)1223 223417

Liver homogenate preparation

Liver homogenates were prepared by adding deionised water to one cerebral hemisphere at a 3:1 v/w ratio in a Precellys CK28 – 7mL tissue homogenising tube (KT03961-11302.7) and subjecting to rapid multi directional motion using a Precellys Evolution homogeniser (Bertin Technologies, Montigney-le-Bretonneux, FR).

LC-MS/MS bioanalytical method

Preparation of calibration standards and quality controls

Calibration (Cal) (0.1 – 100 nmol/L) and quality control (QC) (1, 25, 75 nmol/L) samples were prepared by serial dilution of separate 2 mM DMSO stock solutions in either control plasma or control liver homogenate. Calibration samples were prepared fresh on the day of analysis, QC samples were prepared in advance and stored at -20 ºC alongside homogenised test samples where-by being subjected to the same freeze/thaw cycle.

Bioanalytical Method

On the day of analysis 50 µL of test, Cal or QC sample was subjected to protein precipitation with 150 µL of acetonitrile containing 1 µg/mL of the assay internal standard, a structurally unrelated compound from the AZ compound library. Following mixing and centrifugation 50 µL of the resulting supernatant was transferred to the well of a 96 deep well plate containing 300 µL of water.

50 µL of the resulting extracts were injected onto a gradient UHPLC system (Accela, Thermo Scientific, Hemel Hempstead, Herts, UK) compromising of 0.1% v/v formic acid (aq) as mobile phase A, 0.1% v/v formic acid in methanol as mobile phase B and a Phenomenex Max-RP 50 x 2.1mm column (Phenomenex, Macclesfield, Cheshire, UK) as the stationary phase. Detection was performed using a TSQ Vantage (Thermo Scientific, Hemel

Hempstead, Herts, UK) mass spectrometer operating in selected reaction monitoring mode (SRM) with an electrospray ionisation source (ESI). The mass transitions monitored for compounds dosed orally were the same as detailed in the main manuscript for LESA analysis. Analyte/internal standard peak area ratios were calculated and the calibration data fitted using a linear regression with a $1/x^2$ weighted fit. Test and QC sample concentrations were back calculated from the fitted line. Analytical batches was considered acceptable if 75% of the calibration levels fell within 15% of their nominal concentrations and a minimum of 4 out of 6 of the back calculated QC samples fell within 15% of their nominal concentrations.

LESA optimization experiments

Solvent composition

Figure SI 1 - Graph showing mean intesity of clozapine, albendazole, tamoxifen and astemizole after LESA extraction (n=10) with different solvent compositions of a) Isopropyl alcohol, b) methanol and c) Acetonitrile.

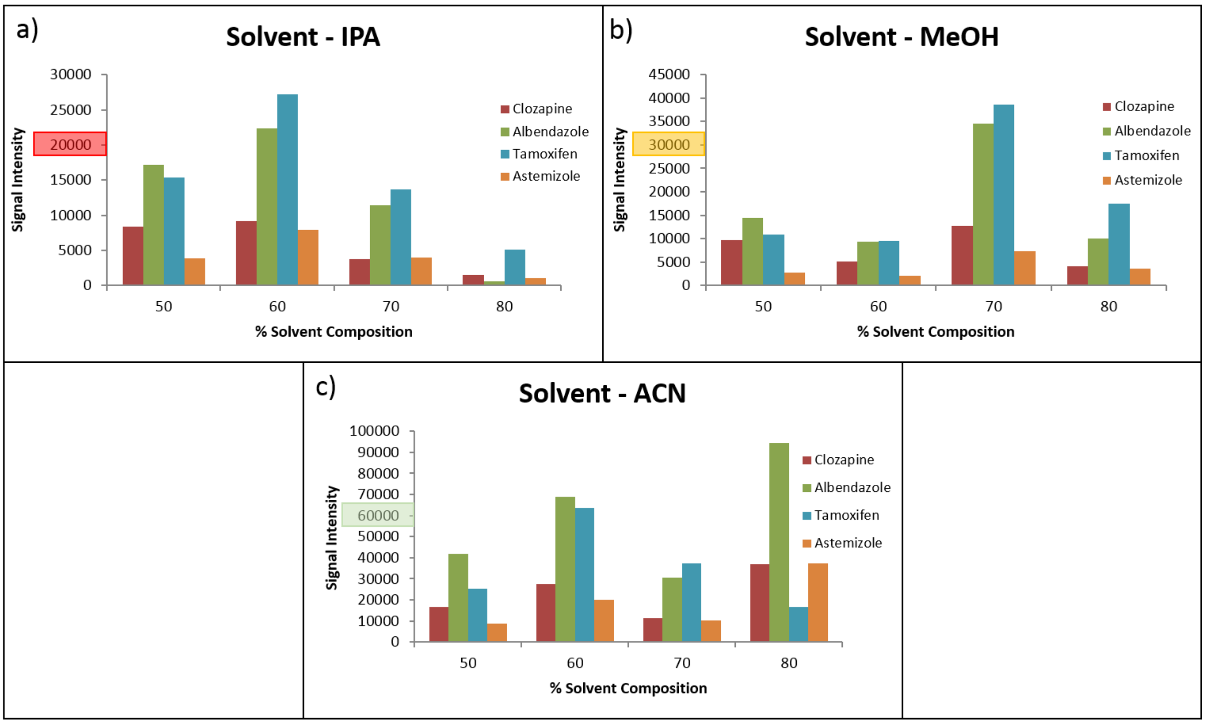

Tissue thickness

Figure SI 2 – Graph showing the effect of tissue thickness on mean signal intensity after LESA extraction (n=10).

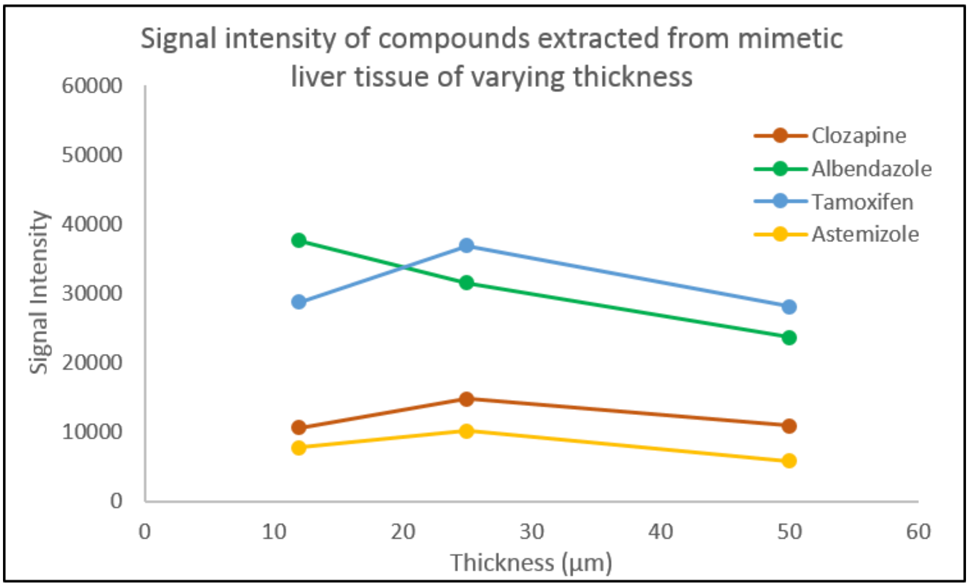

Solvent dwell time

Figure SI 3 – Graph showing the effect of solvent dwell time on mean signal intesity of SCH-23390 after LESA extraction (n=10).

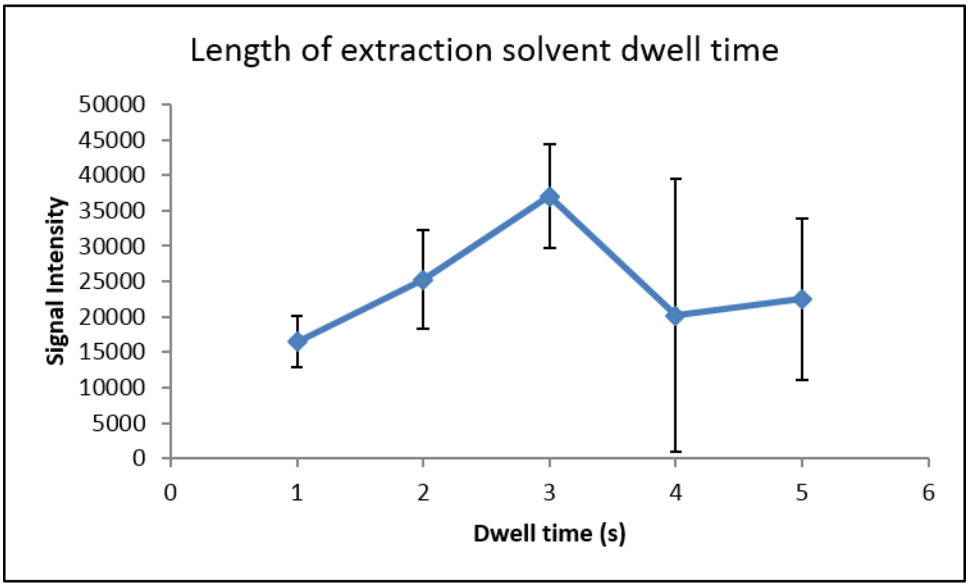

Use of an internal standard

Table SI 1 – Coefficient of variation comparison for tamoxifen, clozapine, albendazole and astemizole after LESA extraction at random points (n=10) on mimetic liver calibration standards with and without an unlabelled internal standard sprayed over the tissue.

Cal Level	Tamoxifen		Clozapine		Albendazole		Astemizole	
	No IS	With IS	No IS	With IS	No IS	With IS	No IS	With IS
0.1	23.2	27.1	33.2	39.8	30.3	36.5	24	28.2
0.5	17.3	27.5	19.9	32.4	11.4	32.4	20.8	34.3
1	8.6	18.6	18.1	33.3	20.2	36.2	17.2	19.5
5	11.7	37.8	28.4	28.5	26.8	24.4	26.6	27.4
10	41.8	42.5	28.6	38.1	23.1	31.1	23.3	35.4
20	23.9	32	21.3	30.5	18.2	29.7	26.1	35.6
50	20.6	23.3	17.7	24.2	15.4	22.6	16.2	21.9
100	25.3	42.5	15	34.6	17.7	35.6	30.2	32.3

Table SI 2 – Coefficient of variation comparison for clozapine after LESA extraction at random points (n=10) on mimetic liver calibration standards with and without (deuterated) clozapine-d4 internal standard sprayed over the tissue.

Cal Level	Clozapine	
	No IS	With IS
0.1	38.8	64.1
0.5	39.9	43.9
1	43.7	46.1
5	19.3	17.1
10	28.7	43.1
20	22.6	31.5
50	44.0	56.5
100	34.7	35.1

Table SI 3 – Coefficient of variation comparison for clozapine after LESA extraction at random points (n=10) on mimetic liver calibration standards with and without (deuterated) clozapine-d4 internal standard spiked into the LESA extraction solvent..

Cal Level	Clozapine	
	No IS	With IS
0.1	12.8	19.7
0.5	40.3	41.1
1	25.4	13.0
5	46.3	31.8
10	41.8	32.7
20	31.1	19.7
50	45.9	38.7
100	31.1	18.6

The reasons for the differences in the Coefficient of variation between different concentrations in tables SI 1-3 are unknown and are likely to be the sum of many contributing factors such as ion suppression, instrument variability and inherent variation in the LESA technique. The differences do not follow a normal distribution.
